# Quality of fish eggs and production of androgenetic and gynogenetic doubled haploids (DHs)

**DOI:** 10.1007/s10695-023-01206-4

**Published:** 2023-06-10

**Authors:** Konrad Ocalewicz

**Affiliations:** Department of Marine Biology and Ecology, Institute of Oceanography, Faculty of Oceanography and Geography, University of Gdansk, Al. M. Piłsudskiego 46, 81-378 Gdynia, Poland

**Keywords:** Egg quality, Androgenesis, Gynogenesis, Irradiation, Transcriptome, Antioxidant enzymes

## Abstract

Induced development of haploid embryos (H) with only paternal (androgenesis) or maternal (gynogenesis) chromosomes requires irradiation of eggs before fertilization or activation of eggs with irradiated spermatozoa, respectively. To provide doubled haploids (DHs), androgenetic and gynogenetic haploid zygotes need to be subjected to the thermal or high hydrostatic pressure (HHP) shock to suppress the first mitotic cleavage and to double paternal or maternal haploid set of chromosomes. Androgenesis and mitotic gynogenesis (mito-gynogenesis) result in the generation of fully homozygous individuals in a single generation. DHs have been utilized in selective breeding programs, in studies concerning the phenotypic consequences of recessive alleles and to evaluate the impact of sex chromosomes on the early ontogeny. Moreover, the use of DHs for the NGS approach radically improves de novo the assembly of the genomes. However, reduced survival of the doubled haploids limits the wide application of androgenotes and gynogenotes. The high mortality of DHs may be only partly explained by the expression of recessive traits. Observed inter-clutch variation in the survival of DHs developing in eggs originating from different females make it necessary to take a closer look at the quality of the eggs used during induced androgenesis and gynogenesis. Moreover, the developmental competence of eggs that are subjected to irradiation before fertilization in order to deactivate maternal chromosomes when undergoing induced androgenesis and exposed to the physical shock after fertilization that leads to the duplication of the zygotes in both mito-gynogenesis and androgenesis may be also altered as irradiation and sublethal values of temperatures and hydrostatic pressure are considered as harmful for the cell organelles and biomolecules. Here, recently provided results concerning the morphological, biochemical, genomic, and transcriptomic characteristics of fish eggs showing high and low competence for androgenesis and mito-gynogenesis are reviewed.

## Introduction

The development of haploid embryos with exclusive maternal (gynogenesis) or paternal (androgenesis) chromosomes requires the activation of eggs with irradiated spermatozoa or irradiation of the eggs before fertilization, respectively. The purpose of the irradiation is a genetic inactivation (damage) of the nuclear DNA in gametes, and both UV rays and ionizing radiation (IR) are used during this step. The duplication of maternal (gynogenesis) or paternal (androgenesis) chromosomes and the production of fully homozygous doubled haploids (DHs) is accomplished by the exposure of the haploid embryos to a chemical or physical (thermal or high hydrostatic pressure-HHP) shock around the prophase of the 1st mitosis, which prevents 1st cell cleavage (Pandian and Koteeswaran 1998). Most of the haploids (Hs) die before hatching due to the disturbed gene expression and impaired body development described as haploid syndrome (Luo & Li 2003). However, haploid embryos represent an excellent source of the haploid embryonic stem cells useful in the developmental research (Araki et al. 2001). Both haploids and doubled haploids have been widely employed in research concerning the phenotypic consequences of recessive alleles (Yi et al. 2009; Kroeger et al. 2014). DH individuals have been utilized in the selective breeding programs (reviewed by Komen and Thorgaard 2007) and in the generation of the isogenic and clonal lines that have been established in several aquaculture and model fish species (reviewed by Franek et al. 2020). The application of completely homozygous DHs improves the de novo assembly of the genomes sequenced using the next-generation sequencing approach (Liu et al. 2012). The homozygosity of the doubled haploids simplifies detailed linkage analyses enabling identification of the chromosome regions connected with the quantitative trait loci (QTL) (Komen and Thorgaard 2007). DH androgenotes and gynogenotes have been used in research related to the sex determination process and morphological differentiation of the sex chromosomes (Ocalewicz et al. 2007; Chen et al. 2009; Michalik et al. 2016). Moreover, androgenesis has been used to investigate the role of the mitochondrial DNA during embryonic development in fish (Brown et al. 2006) and to recover nuclear genomic information of fish populations, lines, or even species from cryopreserved spermatozoa (Babiak et al. 2002).

Both mitotic gynogenesis and androgenesis have been induced to generate DHs in several model fish species including zebrafish (*Danio rerio*) and medaka (*Oryzias latipes*), and many aquacultured marine and freshwater fish species including various species of flatfish, percoids, salmonids, cichlids, and cyprinids (reviewed by Komen and Thorgaard 2007). Unfortunately, the extremely low survival rate of the doubled haploids that rarely exceeds 20% of the hatched fry reduces the wide implementation of mitotic gynogenesis and androgenesis in the aquaculture. In general, the low survival rate of DH individuals is explained by the expression of the deleterious alleles; however, observed in some experiments variation in survival of DH embryos developing in eggs originating from different females suggests a closer look at the quality of eggs utilized during andro- and mitotic gynogenesis would be wise. Moreover, the results of recently performed research show that exposure of eggs to the irradiation and sublethal physical treatment that lead to the production of DHs significantly affects the egg organelles and molecules and reduces their developmental competence. Due to the limited knowledge on the role of the egg quality during the production of DHs, published reviews concerning the chromosome set manipulations, production of doubled haploids, and clonal and isogenic lines in fish have been mostly focused on the technical issues related to the induction of androgenesis and gynogenesis, the efficiency of these processes, the genetic verification of the fish produced, and the application of DHs (Pandian and Koteswaaran 1998; Arai 2001; Komen and Thorgaard 2007; Franek et al. 2020). However, development and better accessibility to the high-troughput omics technologies facilitating molecular characteristics of eggs showing varied developmental competence (Sullivan et al. 2015; Rauwerda et al. 2016; Cheung et al. 2019, Ma et al. 2019, Weber et al. 2021, among others) enable to assess changes in the egg molecules that might appear after exposure to radiation and physical shocks when induced androgenesis and gynogenesis (Gurgul et al. 2018; Ocalewicz et al. 2019). Thus, the main goal of this paper is to present the current state of knowledge on the interindividual variation of the fish egg competence for the DH development and the egg quality and its alteration observed during the induction of androgenesis and mitotic gynogenesis.

## Fish egg–composition and quality

Fish (ovulated) egg is composed of the oocyte nucleus, cytoplasm, and several cytoplasmic organelles, such as the cytoskeleton, mitochondria, cortical alveoli, annulate lamellae, microtubules, Golgi apparatus, and lipid droplets. Moreover, the egg cytoplasm also comprises non-nutritional components deposited during vitellogenesis, including polymerases, chromatin proteins, transcription and translation factors, rRNAs, tRNAs and maternal mRNAs, and nutritive reserves including proteins, amino acids, lipids, carbohydrates, vitamins, hormones, enzymes, and minerals (Lubzens et al. 2010; Bobe et al. 2015). As far as non-nutritional constituents are essential for the fertilization of eggs, the early embryonic development before initiation of the zygotic genome activation (ZGA) during the “mid-blastula transition” (MBT) (Kane and Kimmel, 1993) and for the activation of the zygotic genome (Lindeman and Pelegri, 2010), nutritives are indispensable for the proper further development of embryos (Lubzens et al*.* 2010; Bobe et al. 2015).

The ability of eggs to be fertilized and developed into normal embryos is known as egg quality (Bobe and Labbé, 2010; Migaud et al. 2013). Hence, the production of high quality fish eggs is one of the most important issues in the aquaculture. The egg quality is linked with the yolk formation, and thus, it is influenced by external factors like environmental issues and farming conditions, nutrition, disease prevention, stress, induction of spawning (photoperiod regimes, hormonal stimulation), and husbandry management, including handling and feeding during oocyte maturation (Migaud et al. 2013). The quality of eggs may be also reduced after maturation and ovulation, for example during improper stripping, handling, and storage (Van Eenennaam et al. 2020). Delayed collection and protracted storage before insemination cause so-called post ovulatory oocyte aging impairs the developmental competence of fish eggs and results in reduced survival rate, increase of ploidy anomalies, and larval deformations (Aegerter and Jalabert 2004, Bonnet, Fostier, & Bobe, 2007a, Samarin et al. 2015, 2018, Schreier et al. 2021, Van Eenennaam et al. 2020,). As manipulations performed on the eggs during induced androgenesis and gynogenesis including irradiation (UV and ionizing radiation) and exposure of the chemical or physical shocks have been proved to affect cellular organelles and biomolecules, it must be assumed that both irradiation and physical shocks may decrease the developmental potential of the treated eggs, and therefore, only eggs of the highest quality should be used when mitotic gynogenesis and androgenesis are induced.

Although low-quality fish eggs are characterized by a diminished function of the spindle microtubules (Aegerter et al. 2004), a changed permeability of the egg membrane (Rime et al. 2004), coalesced lipid droplets (Mansour et al. 2007), changed swelling intensity (Lahnsteiner et al. 2001), improper levels of nutritional components (Lahnsteiner et al. 1999), or altered expression of the maternal genes (Aegerter et al. 2005; Sullivan et al. 2015), reliable methods enabling the discrimination of eggs showing low and high quality before fertilization have not been provided so far in most fish species. Application of techniques enabling the quantification of the quality-related alterations in the egg transcriptome may provide molecular markers of the egg quality. So far, maternal transcriptome has been analysed to find differentially expressed genes in eggs showing varied competence for development in several species of fish including rainbow trout (*Oncorhynchus mykiss*) (Aegerter et al. 2005; Bonnet et al. 2007b, Ma et al. 2019; Weber et al. 2021), Atlantic halibut (*Hippoglossus hippoglossus* L.) (Mommens et al. 2010, 2014), Atlantic cod (*Gadus morhua*) (Lanes et al. 2013, Rise et al. 2014), striped bass (*Morone saxatilis*) (Chapman et al. 2014), and sea bass (*Dicentrarchus labrax*) (Żarski et al. 2017). Estimation of the egg quality based the on the level of expression of chosen maternal genes is a promising method, but its application is limited rather to the laboratory research than aquaculture production. However, as the next-generation sequencing approach has been successfully applied to find out transcriptomic differences between low-quality and high-quality eggs (Ma et al. 2019; Weber et al. 2021), it may be also employed to quantify alterations in the maternal RNA in eggs exposed to the irradiation and physical shock during induced gynogenesis and androgenesis.

## Survival of DHs in eggs originating from different females

The most important issue in the generation of DHs is the very low efficiency of mitotic gynogenesis and androgenesis expressed in survival at that particular stage of development. A high mortality of DHs during embryogenesis or after hatching decreases the chances to produce sexually mature DH individuals, which in turn is a limiting factor in the generation of clonal and isogenic fish lines (Franek et al. 2020). In salmonids, use of thousands of eggs to induce mitogynogenesis results in only few females that reach sexual maturation (Jagiello et al. 2018, Hansen et al. 2020). After 4 years of rearing since hatching, four DH rainbow trout females were found but only two of them produced eggs that were successfully used for production of clonal lines (Jagiello et al. 2018). The survival rates of DHs vary between species and within species (reviewed by Komen and Thorgaard 2007). Interfamily variation in survival of the androgenetic specimens has been reported in the brook charr (*Salvelinus fontinalis*) (0–27.9%) (May et al. 1988). Observed variation in survival of DH developing in eggs originating from different females suggests inter-clutch differences in the egg competence for induced androgenesis and mito-gynogenesis. Inter-clutch variation in the survival of the doubled haploids was confirmed in medaka (*Oryzias latipes*) (Naruse et al. 1985), common carp (*Cyprinus carpio*) (Komen et al. 1991), and loach (*Misgurnus anguillicaudatus*) (Arai et al. 2001). In the rainbow trout, a large variation in the survival of DHs developing in eggs originating from different females has been reported in the case of gynogenesis and androgenesis (Table 1). Interestingly, eggs originating from different females that exhibited varied developmental competence for androgenesis and mitotic gynogenesis did not always show a significant variation in survival when fertilized with normal sperm to develop as heterozygous specimens in the control groups in the above-mentioned experiments (Polonis et al. 2019, 2021, Ocalewicz et al. 2020).

**Table 1:** Variation in survival rates (%) of androgenetic and gynogenetic haploid (H) and doubled haploid (DH) rainbow trout (*Oncorhynchus mykiss*) developing in eggs originated from different females

Method	Number of egg donors	Ploidy	Yield (%)			Reference
			Eyed-stage	Hatching	swim-up stage	
Mitotic gynogenesis	15	DH		0–53		Quillet et al. (1991)
	4	DH	3.1–57.2		0.8–41.5	Ocalewicz et al. (2020)
	8	DH	3–42.7	2.1–40.1	1.3–36.9	Ocalewicz (not published)
Androgenesis	4	DH	8.8–15.6		1.1–2.8	Polonis et al. (2019)
	4	H	49.2–59.6	7.2–13.1	0	
	4	DH	1.4–57.1		0.2–27.5	Polonis et al. (2021)
	4	H	3.1–69.1	0–38.3	0	

## Characteristics of eggs showing increased competence for DH production

Despite several prerequisites suggesting that some eggs exhibit higher competence for androgenesis and mito-gynogenesis than others (Quillet et al. 1991, Yamaha et al. 2002, among others), only a limited number of studies focusing on the characteristics of such eggs have been undertaken to date. In the rainbow trout, neither the size of the eggs nor the pH of the ovarian fluid were found to affect the efficiency of the androgenesis (Polonis et al. 2021); however, it has been observed that androgenetic doubled haploids developed better in eggs with uniformly distributed lipid droplets (Polonis et al. 2019). Eggs from the clutch with a significantly increased survival of androgenetic Hs and DHs were also characterized by a significantly increased activity of anti-oxidant enzymes; SOD, CAT, and GPx (Polonis et al. 2019). What is more, the high activity of SOD in eggs with the highest survival of androgenetic embryos persisted also after irradiation. Such an observation may suggest that organelles and molecules including lipids and proteins in some eggs are better protected against IR-generated reactive oxygen species (ROS) due to more efficient cellular anti-ROS mechanisms and that embryos developing in such eggs after exposure to IR may show an improved survival rate.

In another experiment, the transcriptome of eggs originating from four females exhibiting drastic differences in the survival rate of gynogenetic DH embryos ranging from 3 to 57% were examined using RNA-seq and the positive correlation between gynogenetic efficiency and the egg transcriptome profiles was confirmed (Ocalewicz et al. 2020). Eggs from the female that gynogenetic offspring had the highest survival showed an increased expression of 46 genes. The functional analysis of these genes confirmed their engagement in processes related to early embryonic development, cell survival, migration and differentiation, triglyceride metabolism, biosynthesis of polyunsaturated fat, 5S rRNA binding, embryonic neurogenesis, tissue modelling during development, and senescence and aging. The expression of some other genes is connected with cellular components such as the cell membrane and nucleus. One of the genes whose expression was increased in eggs characterized by a high survival of gynogenetic DHs is the *Tert* gene that encodes a protein catalytic subunit of telomerase, an enzyme that maintains the length of the telomeric DNA. Telomerase in some mammals is required for the oocyte development, and the decline in *Tert* expression is paralleled with a decline in oocyte quality (Liu and Li 2010). A significant decrease of *Tert* expression in oocytes is observed during reproductive and postovulatory aging (Yamada-Fukunaga et al. 2013). Moreover, telomerase activity has been confirmed to be important during parthenogenesis (Liu and Li 2010).

The abundance of transcripts of at least some of the genes that show altered expression in eggs with a bigger potential for gynogenesis might be in future considered as molecular markers of gamete competence assuring the higher efficiency of mito-gynogenesis. However, as irradiation and thermal and physical shocks have been found to affect cellular organelles and molecules, even use of the highest quality eggs may not assure a satisfactory efficiency of DH production as these manipulations presumably reduce the developmental competence of the eggs used for androgynogenesis and mitogynogenesis.

## Consequences of egg exposure for UV rays and IR

UV rays and ionizing radiation (gamma rays, X-rays, and higher-energy UV rays) have been used to damage nuclear genome in fish eggs during induced androgenesis (Pandian and Koteeswaran 1998). Both types of radiations act in different ways. UV-irradiation causes dimerization of adjacent pyrimidines resulting in DNA-DNA and DNA-protein crosslinking and chromosome damage. The most harmful lesions that appear in the course of the gamma or X ray irradiation are single-strand breaks (SSBs) and doubled-strand breaks (DSBs) that lead to the chromosome fragmentation. The high penetrating ability and damaging characteristics make IR useful for inactivation of nuclear DNA in the large eggs like those of salmonid fish species (Pandian & Koteeswaran 1998, Komen & Thorgaard 2007). However, exposure of fish eggs to IR in order to damage maternal chromosomes may also reduce quality of the irradiated eggs as IR is harmful not only for the nucleic acids but also for the cellular organelles and macromolecules, including proteins and lipids due to the direct ionization of the biological molecules and indirectly by the radiolysis of water and the generation of free radicals and reactive oxygen species (Hall and Giaccia 2006; Somosy 2000). The pioneering experiments on irradiation of the fertilized loach (*Misgurnus fossilis*) eggs showed that even a low dose of X-rays was damaging for the nucleus and resulted in the arrest of the embryonic development at the late blastula stage, while a higher dose of IR affected the cytoplasm and caused immediate developmental arrest (Neyfakh 1956). In the rainbow trout, radiation doses higher than 65 kR (650 Gy) exhibited a lethal effect and those that ranged from 30 to 50 kR (300–500 Gy) were found to deactivate the maternal nuclear genome with only a minor effect on the embryonic development (Parsons and Thorgaard 1985; Babiak et al. 1998), even though cytogenetic analysis of the rainbow trout androgenetic juveniles and adults that were developing in eggs irradiated with 350 Gy exhibited residues of the irradiated maternal nuclear genome in forms of chromosome fragments (Ocalewicz et al. 2004; Ocalewicz et al. 2010a). Fragments of maternal chromosomes have also been found in the androgenetic brook trout and brown trout (*Salmo trutta*) that hatched from eggs irradiated with 420 and 450 Gy of X rays (Michalik et al. 2014, Michalik et al. 2016). The presence of the radiation-induced maternal chromosome fragments in the androgenetic progenies suggests that doses of X/gamma rays used for the egg irradiation are too low for the complete inactivation of the maternal nuclear DNA. On the other hand, radiation-induced inactivation of the fish gametes may be inefficient due to the DNA repair mechanisms that are active in eggs and spermatozoa (Ocalewicz et al. 2010b, Pei and Strauss 2013). For example, in the UV irradiated gametes, formation of the pyrimidine dimers in DNA can be repaired within the photoreactivation pathway that is a light-induced process mediated by photolyase (Lebeda and Flajshans 2016). To avoid such photo repair reaction, irradiation of gametes and further steps leading to obtain androgenetic and gynogenetic DH zygotes should be completed under darkness.

Radiation-induced chromosome fragments may block cell cleavages and interfere with early embryonic development. Residues of the irradiated chromosomes have been found to alter the formation of the germ cells and cause irregular ovarian development in gynogenetic trout (Krisfalusi et al. 2000). It is not excluded that such fragments may carry highly mutated genetic information dangerous for the carriers at many stages of their ontogenetic development. Moreover, and of equal importance, fish with residues of the irradiated chromosomes are not fully homozygous, which matters when clonal and isogenic lines are generated.

Irradiation applied for inactivation of chromosomes in eggs during androgenesis may be also harmful for the maternally inherited mitochondrial DNA. However, research performed on androgenetic rainbow trout, brook trout (*Salvelinus fontinalis*), and splakes (*S. namaycush* × *S. fontinatlis*) that hatched from eggs exposed to gamma rays show no detectable influence of irradiation on the mtDNA sequences (May and Grewe 1993, Brown and Thorgaard 2006). Also, no damage of the mtDNAs was observed when applied UV rays for the Nile tilapia (Oreochromis niloticus) eggs during androgenesis (Myers et al. 1995). Authors of these reports suggest that mtDNAs are more resistant to irradiation than chromosomal DNA as mitochondrial genome is small, circular, present in large number of copies in egg, and better protected by the double-membrane system surrounding mitochondria (Piko and Matsumoto 1976).

Much lower doses of IR than those used to deactivate the egg significantly increase the generation of ROS and causes oxidative stress. ROSs are thought to be among the factors responsible for the post-ovulatory aging and decrease of the egg quality. Radiation administered during induced androgenesis may also therefore lead to the post-ovulatory aging of irradiated eggs or accelerate this process and affect their developmental competences. Radiation-induced misregulation of mechanisms that govern early development may result in decreased survival of the androgenetic DHs when compared to the gynogenetic DHs that develop in the non-irradiated eggs. Recent studies have shown that haploid brown trout embryos developing in non-irradiated eggs had a significantly higher survival rate than those that developed in irradiated eggs. Among doubled haploids, differences in the survival rates of the gynogenetic and androgenetic specimens also appeared during embryogenesis and deepened as they develop (Michalik et al. 2015). Lower survival rates in androgenotes suggested that eggs’ exposure to IR impairs the developmental potential of the irradiated fish eggs due to changes in the maternal transcriptome what has been verified using standard molecular techniques and the RNAseq approach applied to irradiated and non-irradiated rainbow trout eggs (Ocalewicz et al. 2019). No significant differences in RNA concentrations and integrity were found between eggs treated with 350 Gy and non-irradiated eggs, demonstrating that the exposure of rainbow trout eggs to 350 Gy of IR in order to deactivate maternal chromosomes need not impair the quality or the functionality of the maternal RNA. In the irradiated eggs upregulated expression of transcripts of the immediate early response 2 gene (*IER2*) and the early growth response 1 gene (*EGR1*) were reported. *IER2* and *EGR1* are so-called immediate early genes that are rapidly activated by diverse extra-cellular stimuli including ionizing radiation (Prasad et al. 1995). The EGR-1 protein may reduce so-called induced-radiation resistance and activate pro-apoptotic genes (Ahmed 2004), which may affect early embryonic development of androgenotes.

Ionizing radiation has also been found to affect the cytoskeleton, and doses up to 30,000 rad (50–300 Gy), i.e. doses similar to those administered for enucleation of salmonid eggs for androgenesis, inhibit assembly of the microtubules (Coss et al. 1981). Microtubules are components of the spindle apparatus, cellular structure formed during cell division for the segregation of chromosomes and sister chromatids, and radiation-induced changes in the tubulin may also lead to the chromosome mis-segregation and delay in the cell cycle in the androgenetic early embryos. Observed haploids and haploid/diploid mosaics among androgenetic rainbow trout and brown trout embryos developing in eggs that after irradiation and fertilisation were also exposed to HHP accord with this assumption (Ocalewicz et al. 2010a; Michalik et al. 2015). In such cases, radiation-induced egg deactivation presumably mis-aligned cellular mechanism controlling and delaying the first cleavage making the HHP shock inefficient in aborting the cell cleavage and duplication of the paternal chromosomes. Microtubules also control the movement of the oil droplets (Parker et al. 2014) and radiation-induced alterations in the structure, and the function of the microtubules may impair the cellular mechanism responsible for the active transportation of the lipid droplets. The pattern of distribution of lipid droplets in the rainbow trout eggs before irradiation and after irradiation during induced androgenesis was altered, and an increased ratio of eggs with coalesced droplets was observed after IR treatment (Polonis et al. 2019). This matters since in some salmonids equally distributed lipid droplets assure better embryonic survival (Mansour et al. 2007, Ciereszko et al. 2009).

## Effect of physical shocks applied during androgenesis and gynogenesis on the fish eggs

High hydrostatic pressure and thermal shocks disrupt chromosome segregation by destabilising the spindle microtubules, which is crucial for diploidisation of the androgenetic and gynogenetic haploid zygotes. In fish eggs, however, microtubules also play a role in the transportation of the cytoplasmic particles and factors involved in the processes related to the early cellular differentiation of the blastomeres (Webb et al. 1995). Alterations in the structure of microtubules triggered by the high pressure shock or temperature shock may therefore impair early development of the diploid gynogenetic and androgenetic specimens. In the goldfish and crucian carp (*Carassius auratus* Linnaeus 1758), high pressure shock and heat shock applied to fertilised eggs result in the formation of the thin blastodiscs with poorly developed cytoplasm, delay epiboly, and suppress dorso ventral differentiation. Importantly, dorsal deficiencies in the heat shocked eggs varied among females, which suggested inter-clutch variation in the sensitivity of eggs to the physical shocks that may in turn affect the efficiency of DH production (Yamaha et al. 2002).

Interesting observations have been made in several salmonid fish species when the development of haploids and doubled haploids was induced within the same experiment. Whether it is androgenesis or mito-gynogenesis, until the eyed stage haploid embryos survive much better than their diploid counterparts (Ocalewicz et al. 2010b, Ocalewicz et al. 2013, Michalik et al. 2014, Michalik et al. 2015, Polonis et al. 2018). A mortality rate several times greater in diploid androgenotes and mito-gynogenotes during early embryonic development may be triggered by the mentioned above side effects of the HHP applied in order to duplicate parental chromosomes. Due to haploid syndrome (Lou and Li 2003), only a few of the haploids hatch and none of them survives until the swim-up stage, but further in depth comparative analysis of pressurised and non-treated eggs and early H and DH embryos might help us to understand the cellular and molecular consequences of the application of HHP shock. High values of hydrostatic pressure have been shown to damage DNA-protein structure (Lynch and Sliger 2002) and affect gene expression (Fernandes et al. 2004; Sironen et al. 2002, Jiang et al. 2016), so it may also be harmful to the maternal transcripts in the egg. However, the rainbow trout maternal transcriptome was found to be resistant to a 65.5 MPa of HHP shock of 3-min duration, a standard condition for diploidisation of the trout eggs (Gurgul et al. 2018). No clear evidence for RNA degradation was detected in the pressurised eggs, and analysis of the transcriptome integrity revealed no statistically significant differences between HHP-treated and non-treated eggs. Alterations in the expression profiles of genes related to the development and growth of fish, response to the DNA damage, polymerisation of actin filaments  and action of the spindle microtubules observed in eggs treated with HHP were not substantial. Much greater differences in the maternal gene expression were observed between eggs from different clutches than between HHP-treated and untreated eggs from the same clutch (Gurgul et al. 2018), suggesting that inter-individual differences between rainbow trout females have a greater influence on the egg transcriptome and developmental ability than HHP treatment of the eggs.

## Conclusions

Despite the widespread application of androgenetic and gynogenetic doubled haploids (DHs) in aquaculture and studies related to developmental biology, the efficiency of the generation of fully homozygous DH specimens in fish is restricted. Usually, less than 20% of the androgenotes and mit-gynogenotes tend to hatch, and only few of them survive until sexual maturation, therefore drastically limiting the production of the isogenic and clonal lines of the fish. Most of the mortality observed among DHs results from expression of the lethal alleles, but a huge variation observed in the survival rate of DH individuals in eggs from different females may suggest some female gametes display better developmental competence for androgenesis and gynogenesis than others. Recent morphological, biochemical, and transcriptomic analyses have enabled the characteristics of what may here be referred to as good and bad eggs in terms of the efficiency of induced androgenesis and mito-gynogenesis. The consequences of the exposure of eggs to irradiation and physical shock during androgenesis and mito-gynogenesis have been verified, and alteration in the egg morphology and transcriptome assessed. In some cases, inter-clutch variances between eggs from different females appeared greater than those observed between eggs from the same clutch subjected to extreme external factors and non-treated eggs. This shows the huge maternal influence on the success of the DH production and confirms that poor-quality eggs play a role in the mortality of DH individuals.

## Data Availability

Not applicable.
